# Lack of effects of online HD-tDCS over the left or right DLPFC in an associative memory and metamemory monitoring task

**DOI:** 10.1371/journal.pone.0300779

**Published:** 2024-06-07

**Authors:** Casey M. Imperio, Elizabeth F. Chua

**Affiliations:** 1 The Graduate Center of the City University of New York, New York, New York, United States of America; 2 Brooklyn College of the City University of New York, New York, New York, United States of America; Federal University of Paraiba, BRAZIL

## Abstract

Neuroimaging studies have shown that activity in the prefrontal cortex correlates with two critical aspects of normal memory functioning: retrieval of episodic memories and subjective “feelings-of-knowing" about our memory. Brain stimulation can be used to test the causal role of the prefrontal cortex in these processes, and whether the role differs for the left versus right prefrontal cortex. We compared the effects of online High-Definition transcranial Direct Current Stimulation (HD-tDCS) over the left or right dorsolateral prefrontal cortex (DLPFC) compared to sham during a proverb-name associative memory and feeling-of-knowing task. There were no significant effects of HD-tDCS on either associative recognition or feeling-of-knowing performance, with Bayesian analyses showing moderate support for the null hypotheses. Despite past work showing effects of HD-tDCS on other memory and feeling-of-knowing tasks, and neuroimaging showing effects with similar tasks, these findings add to the literature of non-significant effects with tDCS. This work highlights the need to better understand factors that determine the effectiveness of tDCS, especially if tDCS is to have a successful future as a clinical intervention.

## Introduction

Episodic memory, with its rich contextual details about what was experienced [1], is crucial in daily functioning, and so is our ability to subjectively assess our memory [2]. For example, it may be important to remember what you served a friend at a recent dinner party because if you fail to remember, you might serve the same thing again. However, if you fail to remember, you might nevertheless have a feeling-of-knowing that the dinner menu is in your memory even though you cannot access it. Assessing the contents of memory for a sought-out piece of information is referred to as metamemory monitoring, which can be experimentally indexed in many ways. One way is by using a feeling-of-knowing (FOK) task, in which a judgment is made after a failed retrieval attempt about whether the information is in memory. Episodic memory and metamemory are thought to have both shared and separable brain bases, with overlap in the prefrontal cortex [3–5]. Note that although the medial temporal lobes are the brain areas most classically associated with memory [6,7], since the advent of neuroimaging it has been well known that the prefrontal cortex also plays a role in memory [8–12]. Traditional models of memory and the brain have posited different roles for the left versus right prefrontal cortex, with the right dorsolateral prefrontal cortex (DLPFC) [9,13–16] involved in retrieval and strategic memory searches of episodic memory whereas the left DLPFC was associated with encoding [1,17,18], but subsequent research has also shown that the left DLPFC is involved in retrieval [19–21]. Additionally, both the left and right DLPFC have also been variously implicated in episodic metamemory monitoring [22,23]. Deficits in memory and metamemory occur in certain neurologic and psychiatric disorders (e.g., Alzheimer’s disease, schizophrenia, obsessive-compulsive disorder), and there is interest in testing non-invasive brain stimulation is a potential clinical intervention for declines in memory and metamemory performance [24–27]. Prior work has shown improvements in metamemory monitoring accuracy (i.e., how diagnostic metamemory judgments are of memory performance) when applying high-definition transcranial direct current stimulation (HD-tDCS), a form of non-invasive brain stimulation, over the left DLPFC for a semantic retrieval task [28,29], but did not test episodic retrieval task. Thus, the goal of this experiment was to compare the roles of the left and right DLPFC in metamemory using an episodic retrieval task, and to test if HD-tDCS could improve metamemory monitoring for an episodic task.

### Transcranial Direct Current Stimulation (tDCS)

Brain stimulation, including forms of tDCS, are popular tools in scientific research due to the potential for modulating cognition and potential clinical use [28–32]. TDCS may have greater potential as a scalable intervention compared to other forms of non-invasive brain stimulation (NIBS), such as transcranial magnetic stimulation (TMS), because of its low cost, ease of use, and portability [33,34]. Both conventional and high definition forms of tDCS have been tested as clinical interventions in many different psychiatric [33,35–37] and memory disorders [38,39]. Both conventional and HD-tDCS are types of transcranial electrical stimulation (tES) that rely on sustained weak electrical currents, typically between 1–2 mA. In general, tDCS is believed to modulate the resting membrane potential, thus changing the threshold of excitability of the neurons in the targeted region and inducing cortical plasticity [35,40–42]. In conventional tDCS, two electrodes (anode/positive; cathode/negative) are placed on the participant’s head; current flows from the anode and enters and crosses the brain and then exits out of the brain to the cathode [for a review see—39]. The current flow has effects on the polarity of the neural membrane. When the current flows from outside the neural membrane into it, there will be local hyperpolarization, and when the current flows from inside the neural membrane to outside it, there will be local depolarization. Therefore, whether there is hyperpolarization or depolarization depends on how the neuron is oriented in relation to the electric field generated by tDCS [43–45]. In a typical pyramidal cell, an anode on the scalp will lead to somatic depolarization and apical dendrite hyperpolarization, whereas a cathode on the scalp leads to somatic hyperpolarization and apical dendrite depolarization [46]. When the positive electrode (anode, generating inward current flow) is placed over the target brain region, it is typically referred to as anodal tDCS, whereas when the negative electrode (cathode, generating outward current flow) is placed over the target region, it is typically referred to as cathodal tDCS. When large electrodes are placed relatively far apart on the head, the effects of tDCS can span many brain areas. To increase spatial focality, HD-tDCS was developed, in which multiple, smaller electrodes are positioned to best target the region of interest [42,47,48]. Like conventional tDCS, HD-tDCS can be “anodal” (positive, inward current) or “cathodal” (negative, outward current). One might assume that more focal brain stimulation would result in better performance compared to less focal brain stimulation, but this is not necessarily the case, and may even vary across individuals. In addition to electrode size and placement, another important factor is the amount of current applied. The amount of current applied at the scalp is low in order to minimize adverse events, with the vast majority of studies using 1–2 mA [47,49], which has shown to be safe and tolerable, but there have been experiments using up to 4 mA [47,49,50]. It is worth noting that several studies have tested for linear effects of tDCS on behavior with increasing current applied, and often have shown non-linear effects [51–56]. For example, studies have shown effects of 1 mA tDCS on brain activity or behavior, but not at 2 mA or sham [53,56]. Most studies compare one dosage of tDCS to sham, and there have been effects on cognition at both 1 mA, 1.5 mA and 2 mA [30,32]. When choosing the amount of current to apply, researchers are often guided by prior research showing effects with similar montages.

In addition to arrangement of the electrodes and amount of current, another important factor in tDCS is the timing and duration of the stimulation. tDCS can be described as “online”, meaning that stimulation is applied during the task of interest, or “offline”, meaning that stimulation is applied before the task of interest [41]. Offline stimulation is often used to assess the aftereffects of tDCS, which, depending on the montage, duration and intensity of stimulation, can last up to 2 hours after stimulation for tDCS and HD-tDCS [41,57]. For example, 1 mA of tDCS applied to the motor cortex for 13 min can result in aftereffects that last beyond 110 mins [41]. In sum, there are many parameters that researchers must select when applying tDCS (i.e., anodal vs. cathodal, montage, amount of current applied, duration of stimulation), and these are often driven by prior research successes and/or needs for systematic work.

### Metamemory monitoring and the role of the left and right prefrontal cortex

Metamemory, which is one’s knowledge of the contents of one’s memory [2] is an important aspect of the learning and memory process. Specifically, the ability to assess the contents of one’s own memory for a sought-after piece of information is referred to as metamemory monitoring [58]. Metamemory monitoring can be assessed at encoding or retrieval [2] with different measures being employed depending on the time of assessment. The current study focuses on metamemory monitoring at retrieval, and utilizes FOK ratings, which are prospective judgments about being able to later retrieve an unrecallable piece of information [59]. FOK ratings are given on a scale, such as a scale ranging from 0% sure of recognizing the correct answer to 100% sure of recognizing the correct answer, and often correlates with objective memory performance [59]. The extent to which FOK ratings are associated with the individual’s memory (i.e., higher ratings given for correct recognition, lower ratings given for incorrect recognition) is called metamemory monitoring accuracy, which can be assessed using a variety of indices, including correlational methods such as Phi and Goodman Kruskal’s gamma coefficient [60], and measures based in signal-detection theory, such as meta d’ and d_a_ [60,61]

There has been a longstanding interest in the role of the prefrontal cortex in metamemory since neuropsychological studies showed that the prefrontal cortex, broadly speaking, has a role in FOKs [62–64]. Patients with Fronto-Temporal dementia [65], frontal lobe damage [62,66,67], and normal aging [68,69] have all shown impairments in FOK. However, many of these individuals also showed poorer memory performance, which may have influenced their FOK judgments. Functional magnetic resonance imaging (fMRI) studies have also shown that activity in the PFC correlates with different aspects of FOK, such as the process of making metamemory judgments, the level of FOK or particular FOK rating expressed, and FOK accuracy [5,22,23,66,70]. The process of making FOK judgments showed greater activity in the vmPFC, DLPFC, and other regions compared to a recognition task [3]. Turning to the level of FOK ratings expressed, many studies have shown that activity in prefrontal regions modulates based on FOK ratings [23,70–73] but there is also variation based on the stimuli used, with regions in the parietal and temporal cortices also showing modulation by the level of FOK depending on the task [3,21,23,71,73]. Initial work on FOK ratings examined FOK as an intermediate state between successful recall and failed recall, showing intermediate levels of activity in left and right PFC, left posterior parietal cortex and the anterior cingulate cortex [23,70]. Other research showed linear increases in activity as FOK ratings increased in bilateral ventral, dorsal and anterior prefrontal regions, and medial prefrontal cortex in a general knowledge task [70] and famous faces and names task [21]; additionally, there were linear increases in left parietal cortex, bilateral superior temporal, and right anterior temporal regions for a famous faces and names task as FOK increased [21]. Few fMRI studies have investigated FOK accuracy, but Schnyer et al. [66] showed activity in the VLPFC, vMPFC, ACC and MTL correlated with FOK accuracy. Overall, there is evidence that the prefrontal cortex is involved in FOKs, but other regions are as well.

Based on the prevalence of PFC activity associated with FOKs from the neuroimaging [5,22,23,66,70] and neuropsychological data [62–64], studies have begun to use non-invasive brain stimulation to test causal role of the DLPFC in the feeling-of-knowing [28,29,74]. Earlier work showed that 2 mA of anodal HD-tDCS over the left DLPFC led to increased FOK accuracy for a semantic memory task (i.e., general knowledge retrieval) [24,25]. Subsequent work tested whether or not there would be similar findings using conventional tDCS, but showed no effects of 2mA of conventional, remotely supervised effects of anodal tDCS over the right or left DLPFC on FOK in a semantic metamemory task [75]. Similarly, there were no effects of 2mA of conventional, remotely supervised effects of anodal tDCS over the right or left DLPFC on FOK in an episodic metamemory task [75]. Taken together, these findings raise two possibilities: 1) 2 mA of anodal HD-tDCS is effective at inducing changes in metamemory, whereas conventional tDCS is not, or 2) 2 mA of anodal HD-tDCS is effective at inducing changes in metamemory for a semantic task, but not an episodic task. Therefore, the next step in understanding the ability of tDCS to alter metamemory requires investigating the effects of 2 mA of anodal HD-tDCS on metamemory for an episodic task. No work, to our knowledge, has investigated effects of cathodal tDCS over the DLPFC on metamemory, and thus, could be an area for future research as well. It is worth noting that one study used theta burst stimulation (TBS), a different form of NIBS, and targeted a more superior area of the PFC (BA 9/10) and showed significantly higher FOK accuracy for TBS over the right PFC compared to the left PFC [74] in a visual associative memory task. Thus, there is some evidence that NIBS targeting the right PFC can improve episodic FOK accuracy, but it is unknown if these findings will extend to HD-tDCS.

### Episodic memory and the role of the left and right prefrontal cortex

Although our primary interest was in metamemory, metamemory cannot be considered without attention to the primary memory task. Episodic memory is the ability to consciously recollect a prior experience, and often includes rich contextual details surrounding that experience [1]. Because “binding” information is a crucial part of memory processes, many laboratory studies of episodic memory use associative memory tasks [76], in which participants are given pairs of unrelated stimuli and are asked to encode the pair, and then are tested on the pair at a later time [76,77]. Test format varies, but often participants are shown one of the paired associates (cue) and then asked to retrieve (either via recall or recognition) the item that was previously paired with the cue (target).

Neuroimaging studies of episodic memory have long shown prefrontal activity associated with episodic retrieval [1,78,79]. The DLPFC has been associated with different aspects of retrieval including being in a retrieval mode [80–82], post-retrieval monitoring and evaluation [20,83–86], general decision operations [87–89], and control-related modulation of the hippocampus [90–92]. Much of the fMRI work focusing on understanding the role of the PFC in retrieval has examined the right DLPFC because it showed more robust activation in earlier studies [1,13–17,80,81], but a meta-analysis of fMRI studies of episodic memory also show the left DLPFC has a role [93]. Whether or not recruitment of the DLPFC is more lateralized may depend on tasks specifics, such as stimulus types with left DLPFC activity associated with post-retrieval monitoring for verbal stimuli [20] and right DLPFC for visual stimuli [84]. Other work has shown bilateral involvement of the DLPFC with controlled retrieval and modulation of the hippocampus [90].

Non-invasive brain stimulation, such as transcranial magnetic stimulation (TMS) and tDCS, have been used to test the causal role of the left vs right PFC in memory based on correlational findings from fMRI data. For TMS, different factors, such as the pattern of stimulation determines whether TMS is disruptive of facilitative [94–96]. Multiple TMS studies have shown that the right DLPFC plays a role in episodic retrieval [74,97–100], but there has been evidence for a role of the left DLPFC in episodic retrieval as well [19]. One possible explanation for hemispheric differences related to strategy use, with one TMS study indicating that the right DLPFC may be involved during the use of a retrieval strategy that employs cognitive control, whereas the left DLPFC may be more involved in retrieval when no specific strategy is being utilized [19].

Although there is strong evidence about the causal role of the DLPFC in episodic retrieval from TMS [19,97,99–101], the results from tDCS have been varied [32,102–108], and have mostly used conventional tDCS rather than HD-tDCS. Note that many tDCS studies focus on encoding [104,107,109–113], but focusing on retrieval is most relevant for the current work. Looking at studies that examine tDCS at retrieval, some studies showed that anodal [102,106,107,114,115] and cathodal [105] DLPFC stimulation targeting retrieval improves episodic memory, while others show no improvements with anodal [103,116,117] or cathodal [102,118] stimulation [for a review see—113]. tDCS studies focusing on retrieval have shown that both online [106,115] and offline [105,107,114] tDCS over the DLPFC can result in improved episodic memory. The majority of this work has examined item recognition [102,105,106,115] or recall [108,114,119], with fewer studies using associative tasks [but see—105,109]. These studies use a variety of tasks, montages, timing, and amount of current applied, which can make it difficult to easily assess when tDCS is effective at altering memory. At a minimum, it shows that effects of tDCS may be nuanced, and many parameters need to be considered. More concretely, De Lara et al. [103] used a verbal associative learning task and showed no effects of online anodal tDCS over the left DLPFC on retrieval. In contrast, Gray et al. [107] showed offline anodal tDCS over the left DLPFC improved recollection using a difficult source memory task. However, a series of follow up experiments showed that improvements in episodic recollection accuracy are time-of-day dependent, which could explain some of the null results other work has shown [32]. Recent work has also shown that online anodal tDCS over the right DLPFC in a proverb-famous name task increased recognition, but this depended on task order [75]. Although there have been many studies on episodic retrieval using conventional tDCS to target the DLPFC, fewer studies have used HD-tDCS and often those had different targets. For example, Huang et al., [120] used HD-tDCS to target anterior versus posterior default mode network (DMN) regions and showed better recollection for Swahili-English word pairs anodal HD-tDCS targeting the posterior DMN and cathodal HD-tDCS targeting the anterior DMN compared to sham. Thus, more work that allows a systematic examination of the role of the left and right DLPFC in associative retrieval and the ability of HD-tDCS to improve associative retrieval is of interest, in addition to our primary interest in metamemory.

### The current study

The main goal of the current study was to test the causal role of the DLPFC in FOK using an episodic memory and metamemory task using HD-tDCS. This was motivated by prior work showing HD-tDCS induced improvements in metamemory accuracy for a semantic task [28,29] and interest in testing whether or not this extended to an episodic memory and metamemory task. We chose to apply 2 mA and focus on online HD-tDCS because previous work showed that HD-tDCS over the left DLPFC led to improvements in metamemory monitoring accuracy [28,29] and retrieval in a semantic memory task [28]. We modeled our task after the task used in an fMRI study by Reggev and colleagues [22] because they used episodic and semantic tasks that were similar in structure to examine shared and distinct neural correlates of metamemory; participants studied novel proverb-famous name pairs in an associative encoding task, attempted to recall the name associated with the proverb, made a subjective FOK judgment, and then completed a forced choice recognition task. This experiment used a within-subjects design, and during the retrieval phase, participants received online left anodal DLPFC, right anodal DLPFC, or sham HD-tDCS. Based on prior research [74], we expected that online anodal HD-tDCS over the right DLPFC during retrieval would lead to increased memory performance compared to anodal stimulation over the left DLPFC [1,80–82]. Turning to metamemory monitoring, given that both left and right DLPFC have shown involvement in FOK ratings [21–23], and our overall prediction was that stimulation of both the left and right DLPFC would lead to increased magnitude of FOK ratings, and we did not expect to see a difference in FOK ratings for online HD-tDCS over the left versus right DLPFC. Further, we predicted that online HD-tDCS over the left DLPFC would improve metamemory monitoring accuracy compared to sham, similar to a semantic task [28,29].

## Materials and methods

### Participants

To determine the sample size, a power analysis was conducted via G*Power software utilizing parameters (i.e., effect size, power) from prior HD-tDCS work [29,31,75,121]. This analysis determined that 28 participants were needed to obtain a medium effect size, with an alpha of 0.05 and 80% power [122]. However, due to the counterbalancing of the test versions and stimulation conditions, 36 participants were needed and determined to the final sample size. Data collection continued until there was complete data from 36 participants, as indicated by the power analysis.

Forty-one participants originally participated in this cross-over, single-blind, sham controlled experiment, though, as per the power analysis, the final sample consisted of 36 participants, counterbalanced for stimulation type and test version. The experiment was single blind, such that the experimenter was aware of the stimulation condition and knew the demographic information for the participant. Recruitment began in October of 2019, paused due to COVID-19 restrictions from March 2020 to September 2021, and completed in September of 2022. Of the 41, one participant dropped out because they were unable to commit to the time required for the experiment, one because they could not tolerate the stimulation, one because the researchers were unable to achieve satisfactory electrode contact quality due to their hair style, and two were unable to complete their sessions because of the COVID-19 shutdown. Though this may seem like a large attrition rate, two of the participants were removed before even participating (i.e., could not tolerate stim and hairstyle), and two participants could not complete the experiment because of the COVID-19 pandemic, which affected everyone and should not be counted as attrition due to the experiment. Thus, with the one participant who dropped out due to time commitment, our attrition rate was 7.7%. During data collection, participants were assigned a unique subject code to de-identify their data. However, because the researchers were in contact with participants for the experimental sessions and during data collection, researchers had access to identifying information for participants. After data collection was completed, data were analyzed using subject codes and links to any identifying information were not viewed. For the final sample of 36 (*F* = 30, *M* = 5, *Other* = 1) the ages ranged from 18–34 (*M* = 21.69, *SD* = 3.82). Of these participants, 24 identified as not Hispanic/Latinx and 12 identified as Hispanic/Latinx. Sixteen of the participants identified as Asian, 1 as Native Hawaiian, 6 as Black or African American, 5 as White, and 8 identified as mixed race or other.

### Ethics statement

The procedures in this experiment were approved by the Human Research Protection Program at the City University of New York (CUNY). Participants gave written informed consent at the beginning of their experimental sessions and were informed that they could withdraw at any time without loss of benefits. Informed consent was witnessed by a trained research assistant, and participants were given the option to: 1) share their de-identified data with other researchers for future research (n = 24), 2) share their de-identified data for future research in the lab (n = 22), or 3) only have their de-identified data used for the current experiment (n = 3), or did not make a selection (n = 11).

### Behavioral task

**Fig 1:**
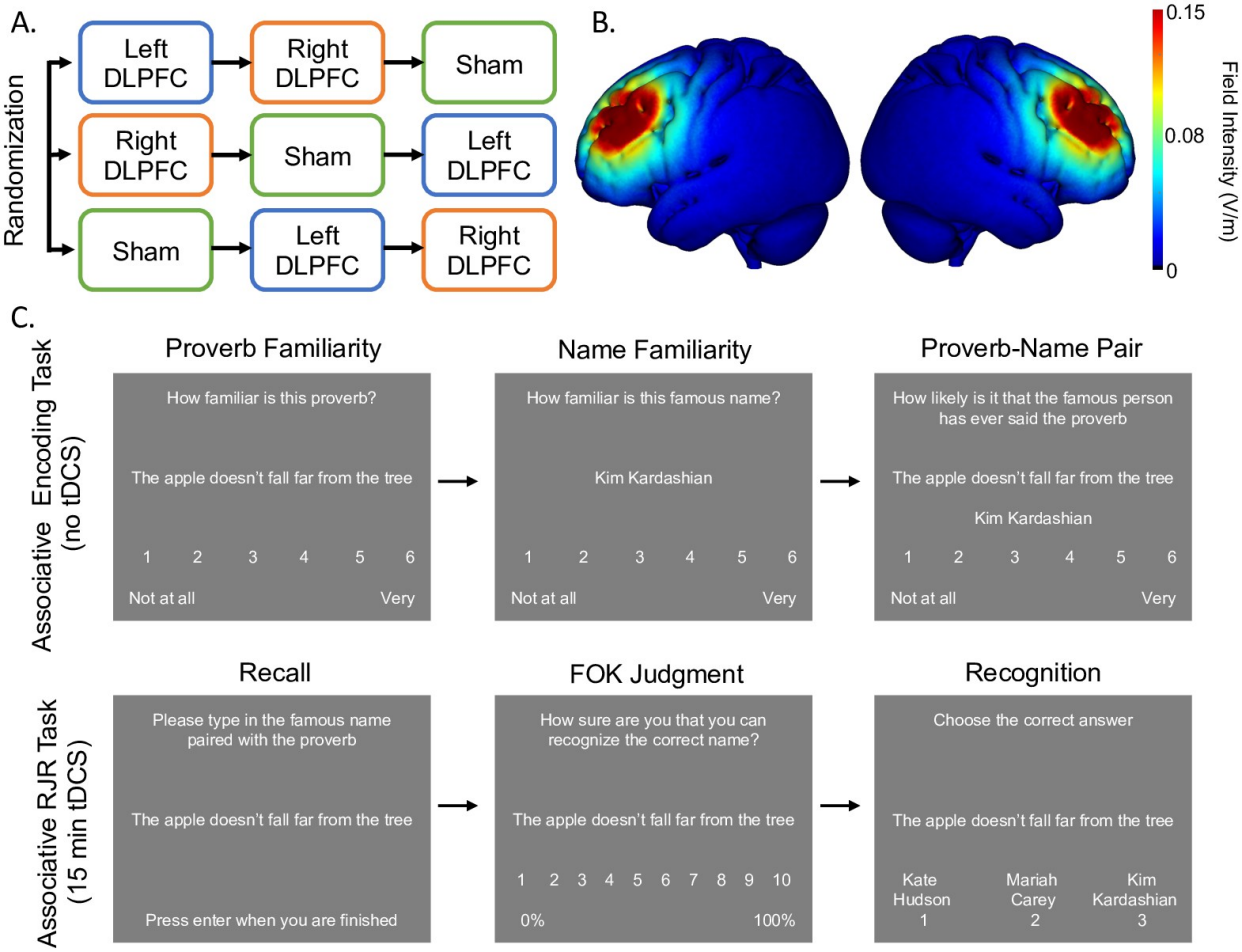
Overall task design (A), current intensity map (B), and behavioral task (C). Participants received three types of stimulation, at least 1 week apart, and were randomized to a specific order in a cross-over design (top, left). In addition to sham HD-tDCS, participants also received anodal HD-tDCS over the left and right DLPFC. Maps generated using HD-Explore (Soterix Medical, Inc.) depict the predicted field intensity (V/m) in the prefrontal cortex (top, right). For the behavioral task (bottom), example stimuli are shown for the multi-part encoding task, which consisted of rating proverb familiarity, name familiarity, and the likelihood the famous person ever said the proverb. Example stimuli are also shown for the Recall-Judgment-Recognition task, during which participants received HD-tDCS.

Participants came to the lab for three separate visits, each at least one week apart and at approximately the same time of day. Before the session, participants were randomly assigned to a counterbalanced order of three test versions (A, B, or C). The proverb-name memory task was modeled after work by Reggev and colleagues [22] in which proverb name pairs were utilized in an episodic memory and metamemory task. The current study utilized lists of proverb/famous name pairs that were normed for our population of interest. Both the proverbs and famous names were chosen to be culturally specific to our population of interest. The test versions each contained a unique set of 100 previously unrelated proverb/famous name pairs (i.e., these were randomly assigned pairs, not quotes by the famous person), which were presented during the sessions (i.e., “Against the grain” was paired with “Whitney Houston”). Proverbs and famous names were chosen and assigned to three lists matched on number of words in the proverbs, number of letters in the famous names, number of letters in the proverb/name pairs, and on familiarity ratings (1 = Not at all familiar, 5 = Extremely familiar) from an online pilot study with 68 Brooklyn College students. These lists did not change throughout the study but were counterbalanced for visit order. Participants underwent encoding, as well as memory and metamemory testing for each pair (see Fig 1C). Participants completed a practice test for each part of the task to ensure that they fully understood the instructions. During encoding, participants first rated the familiarity of the proverb using a scale of 1 (not at all familiar) to 6 (very familiar). Next, they rated the famous name using the same scale. Finally, they rated how likely it is that the famous person has ever said the paired proverb, using a scale of 1 (Not at all likely) to 6 (Very likely). Each proverb, name, and pairing stayed on the screen for 3 seconds, with a 1 second fixation cross between each presentation. Participants were instructed to read the stimuli silently and attempt to remember the pairing in an intentional encoding task. The encoding phase lasted approximately 25 minutes. The goal of the familiarity ratings was to ensure participants to paid attention to the proverb, the name, and then the pair so that they would be able to better encode this information.

After encoding, HD-tDCS stimulation began, participants were given a Recall-Judgment-Recognition (RJR) task for all 100 pairs. The RJR task is traditionally used in FOK studies [59] and was the task used in prior work showing effects of HD-tDCS on metamemory accuracy [28,29] that we sought to extend with the current study. First, participants were shown the proverb alone and were asked to recall the associated name and type it in. If they could not remember the previously paired name, they were instructed to type in “idk” for “I don’t know”. Then, participants gave an FOK rating, in which they indicated how likely they thought they were to recognize the answer to the question at a later time, on a 1 to 10 scale, with 1 representing feeling 0–10% sure in their ability to recognize the correct answer and 10 representing feeling 91–100% sure in their ability to recognize the correct answer, and the other numbers representing 10% increments of increasing certainty. This rating scale has commonly been used in FOK studies [29,31,75,121] and utilizes percentages as anchor points to give the participants a concrete interval between each Likert point. Finally, participants completed a 3 item (1 correct, 2 incorrect) forced choice recognition test for the proverb/name pair, in which they were shown the proverb and asked to select the previously paired name. The 2 incorrect names were previously paired with other proverbs, ensuring that participants could not use name familiarity alone for the associative recognition task. Two incorrect choices were used because pilot work with these proverb/famous name lists showed that the task 4 answer choices (1 correct, 3 incorrect) was too difficult, and performance was at floor. Thus, we utilized a 3-item forced choice recognition test for the current study. Participants did not receive feedback for their answer choices.

### HD-tDCS method

Before the first session, participants were randomly assigned to a condition schedule, which incorporated two active stimulation conditions (active left DLPFC and active right DLPFC) and one sham HD-tDCS condition (see Fig 1A). For sham HD-tDCS, half the participants received sham HD-tDCS over the left DLPFC, and the other half received sham HD-tDCS over the right DLPFC. This was counterbalanced in accordance with stimulation order, as well as test version in a latin square design.

HD-tDCS was administered using the Soterix 1 x 1 tDCS Low-Intensity Stimulator (Model 1224-B, Soterix Medical, New York, NY) with a Soterix 4 x 1 adaptor. Electrodes were sintered Ag/AgCl ring electrodes with an outer radius of 12mm and inner radius of 6mm. Electrodes were fixed on an EEG cap via HD electrode holders (Soterix Medical, New York, NY), and electroconductivity was ensured by filling the holders with Signa Gel. The “anode” was set to deliver a total current of 2 mA, which has been shown to be a safe and tolerable current for HD-tDCS [47,49,123]. Return electrodes were set to share 2 mA of current, with each electrode receiving 0.5 mA. Active and sham HD-tDCS montages that targeted the left DLPFC placed the stimulating electrode (“anode”) at the F3 position, and the four return electrodes (“cathodes”) placed at AF3, F1, F5, and FC3. Active and sham HD-tDCS montages that targeted the right DLPFC placed the stimulating electrode (“anode”) at the F4 position, and the four return electrodes (“cathodes”) placed at AF4, F2, F6, and FC4. Electrode positions in these montages were based on prior work targeting these regions [28,29], as well as simulated current maps created via computational models using HD-Explore (Soterix Medical, New York, NY) that showed good current flow in the left and right DLPFC (See Fig 1B).

To ensure proper contact quality, stimulation was not initiated until all electrode impedances were below 1.5–2 contact units, with no one electrode having more than double the contact quality of any other electrode (i.e., if one electrode had an impedance of.60, then no electrode could have an impedance of 1.20 or higher). For average impedance by time point recorded, see Table 1. Current ramped up to 2 mA over 30 s and was delivered for 15 min during the RJR task, which was the approximate length of the task. Note that the device used did not have a timer for 15 min, so the device was set for 20 minutes and manually aborted after 15 min. As described in the Soterix 1 x 1 manual [124], manually aborting the stimulation ramps the current down to zero at a safe pace (30 s) and terminates the stimulation run. This method is suggested as the standard for aborting stimulation before the time is completed and is considered safer than turning off the device.

**Table 1:** Average impedance values and 95% confidence intervals (CI) across electrodes by stimulation (Stim) condition and time point recorded.

	Before Prestim Tickle	95% CI	After Prestim Tickle	95% CI	Before Stim	95% CI	After Stim	95% CI
Left DLPFC	.80	[.64 -.95]	.55	[.47 -.64]	.50	[.42 -.58]	.21	[.19 -.23]
Right DLPFC	.78	[.61 -.96]	.55	[.45 -.66]	.52	[.42 -.63]	.20	[.18 -.23]
Sham	.80	[.65 -.95]	.58	[.48 -.68]	.58	[.41 -.75]	.41	[.34 -.49]

**Fig 2:**
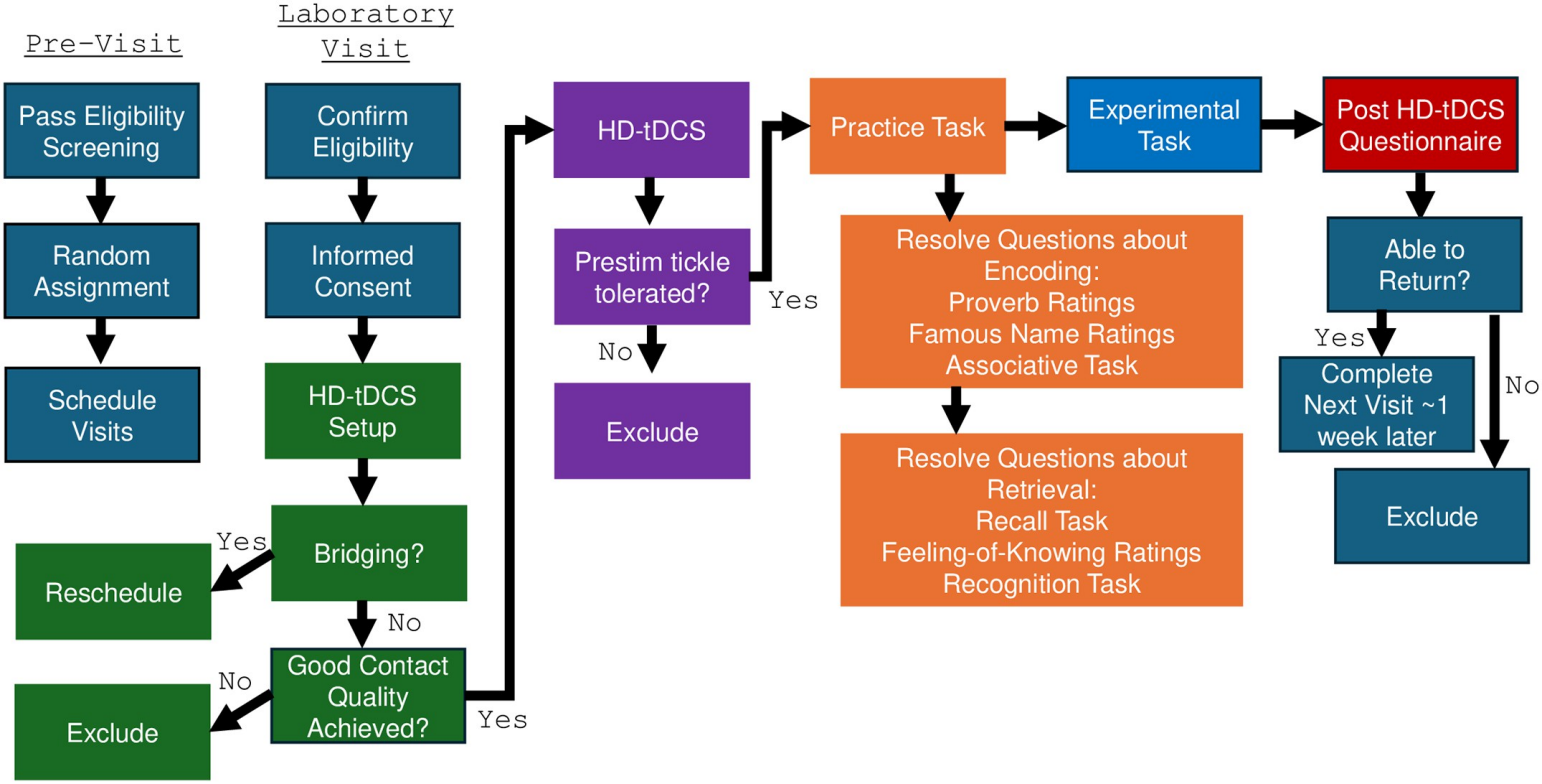
Flowchart detailing the experimental procedure that participants experienced. First, participants were given eligibility screening and if they passed the screening, they were randomly assigned to a task and stimulation schedule, and their visits were scheduled. Next participants came to the lab for their first visit, where their eligibility was confirmed, and they were consented. After consenting, HD-tDCS setup was initiated, and if there was no bridging and good contact quality was achieved, participants moved on to the pre-stim tickle. If they tolerated the pre-stim tickle, a practice test for each phase of the experiment was given, and during this time researchers resolved any questions that the participant may have had. Participants then did the experimental task, followed by a post HD-tDCS questionnaire. If they were able to return, they completed their next visit at least one week later, and started the process again with HD-tDCS setup.

For sham HD-tDCS, the current ramped up to 2 mA for 30 s, then ramped back down to 0.01 mA, and stayed at this current for the remainder of the 15 min, and then ramped down to zero when the abort button was pressed. During the initial session, participants received a “pre-stim tickle”, in which the current ramped up to 1 mA for 30 s, then ramped back down to 0 mA, to ensure that they could tolerate stimulation. The pre-stim tickle was also applied during the subsequent sessions to keep all sessions as consistent as possible. As with prior work [28], at the end of each HD-tDCS session, participants reported tDCS sensations and perceived stimulation condition (i.e., active or sham) by filling out a questionnaire in which they were asked if they experienced any of the following side effects of HD-tDCS: headache, neck pain, scalp pain, tingling, burning, skin redness, sleepiness, trouble concentrating and acute mood change [42]. They were asked to rate the severity of the side effects on a scale of 1 (absent) to 4 (severe), as well as rate their perception of the relationship between each sensation and stimulation on a scale of 1 (none) to 5 (definitely). Finally, at the end of the questionnaire, participants were asked to indicate if they believed that they received active or sham stimulation. Despite some reports of “severe” side effects, and participants being informed that they were free to stop stimulation and the experiment at any time, all participants who tolerated the “pre-stim tickle” continued with the experiment, regardless of sensation reporting. For a full workflow of the experimental sessions, please refer to Fig 2.

### Data analyses

To ensure that variability in participant reactions to HD-tDCS did not influence the results, differences in sensations, tolerability, and blinding of HD-tDCS between stimulation conditions were tested using Friedman and Cochran’s Q tests, respectively. Only sensations that were attributed to the HD-tDCS were analyzed (e.g., trouble concentrating that was not indicated as being related to HD-tDCS was analyzed as absent) to ensure that only stimulation related sensations were analyzed. Prior to analyzing the behavioral data, two researchers checked the free recall answers for correctness. Any discrepancies were resolved by Researcher 1 (CI). Cohen’s kappa was used to assess interrater reliability and showed that the researchers agreed 98% of the time. Also, before examining metamemory monitoring and control, we tested whether memory performance was matched across test version and visit order using Bayesian repeated measures ANOVAs on recall and recognition. There was a difference in recall performance by test version, thus data used in subsequent analyses concerning recall were standardized by test version to account for test version performance. Half of the participants received sham over the *left DLPFC*, while the other half received sham over the *right DLPFC*. To ensure that there were no differences in memory and metamemory performance between sham montages, several Bayesian independent samples t-tests on our memory and metamemory measures.

To assess how online HD-tDCS over the left and right DLPFC affected memory and metamemory performance, as compared to sham, we conducted several Bayesian repeated measures ANOVAs on average recall performance, recognition performance and metamemory monitoring accuracy, with stimulation as a factor. It should be noted that the experiment was not designed to test recall. In fact, recall performance was intentionally low because FOK ratings are defined as occurring for items that are not recalled [59]. Due to the nature of FOK ratings, average FOK ratings and metamemory accuracy were calculated using only the items that were incorrect at the initial recall [28,29]. We first tested the effect of stimulation on FOK ratings, and whether this differed by recognition accuracy. Then we tested formal measures of *metamemory accuracy*, which indexes how well the subjective FOK ratings predict objective memory performance on a trial-by-trial basis. In this experiment, recognition was the measure of memory performance. To compute metamemory monitoring accuracy, d_a_, a measure based in signal detection theory [28,29,61] was used. We chose to use d_a_ because it is has been shown to be a superior measure for evaluating manipulation effectiveness on metamemory accuracy for both within and between groups compared to other metamemory monitoring accuracy measures such as gamma [61]. To calculate d_a_, the following formula was used, in which y_0_ is the y-intercept and *m* is the slope of the normal-deviate isosensitivity function:

$$d_{a}=\frac{\sqrt{2}y_{0}}{\sqrt{1+m^{2}}}$$

Bayesian analyses were used to show if our data favored the null hypothesis over the alternative, or if our protocol was not strong enough to show evidence for the alternative hypotheses [125]. Bayesian statistics differs from conventional significance testing, because, unlike a p-value, which only evaluates the data given the null, bayes factors evaluate the data under both the null and alternative hypotheses [126]. In other words, bayes factors compare how likely the null is or is not compared to the alternative hypothesis, given the data and prior research [125]. For example, a frequentist null hypothesis evaluates if the mean difference between groups is statistically different from zero, whereas a Bayesian null looks to show if there is any mean difference between the groups [127]. Bayesian analyses were conducted using JAMOVI software with the default Cauchy priors and all analyses were compared to the null model [128]. The researchers chose to use the null model compared to the best model because the frequentist analyses originally conducted showed no significance, and therefore wanted to compare our data to the null model. Bayes factors (BF) were interpreted according to Lee and Wagenmakers’ classification scheme for reporting Bayes factors [129].

## Results

### Stimulation sensations and blinding

Most participants tolerated HD-tDCS well and only experienced mild to moderate side effects that were attributed to HD-tDCS, and no side effects were rated as “severe” (See Table 2). Friedman tests showed that participants rated the *left DLPFC* (*M* = 1.39) as having more burning sensations compared to both the *right DLPFC* (*M* = 1.08) and sham (*M* = .75); *X*^*2*^(2) = 10.381, *p* = .006. For all other sensations, there were no significant differences between the stimulation conditions (all *p’s* >.05).

**Table 2:** Stimulation sensations (collapsed across “mild” and “moderate” ratings) attributed to HD-tDCS and blinding.

Sensations	Left DLPFC	Right DLPFC	Sham
headache	4	3	3
neck pain	2	2	2
scalp pain	11	10	7
tingling	32	27	29
burning	22	17	12
skin redness	2	1	1
sleepiness	18	15	15
trouble concentrating	17	12	15
mood changes	4	1	1
other	0	2	0
**Blinding**			
**“Active” Guesses**	28	24	19

To assess participant blinding to HD-tDCS conditions, a Cochran’s Q analysis was conducted on participants guesses about receiving “active” stimulation [130]. Specifically, we assessed what the participants perceived the stimulation to be, not whether their guess was accurate or not. There were no significant differences in guessing “active” between any of the stimulation conditions: χ^2^(2) = 4.083, p = .130. This shows that participants did not perceive stimulation to be “active” more for the active conditions (i.e., left and right DLPFC) compared to sham.

#### Tests of order effects and test version

A Bayesian RM ANOVA on average recall performance by visit order showed moderate support for the null hypothesis that there were no mean differences in proportion correctly recalled between visits (BF_10_ = .261; P(M|data) = .207; 95% Credible Interval (CI) [.04,.09]). Our data was 3.83 times more likely under the null hypothesis compared to the alternative. However, unlike in pilot testing, test version did play a significant role in recall accuracy, with our data showing moderate support for the alternative hypothesis that there was a significant difference in proportion correctly recalled by test version (BF_10_ = 8.109; P(M|data) = .889; 95% CI [-.03,.09]). Follow up Bayesian paired samples t-tests were conducted to determine which test version participants performed differently on. The data showed moderate support for the alternative hypothesis that there was a mean difference in the proportion correctly recalled between test versions A and B (BF_10_ = 6.842; 95% CI [.02,.12]), with lower proportion correctly recalled on version B (*M* = .05, *SD* = .05) compared to version A (*M* = .08, *SD* = .08). There was also anecdotal support for the alternative hypothesis that there was a mean difference in the proportion correctly recalled between test versions B and C (BF_10_ = 2.025; 95% CI [.03,.10]), with a lower proportion correctly recalled on version B compared to version C (*M* = .07, *SD* = .08). The data showed moderate support for the null hypothesis that there was no mean difference in proportion correctly recalled between test versions A and C (BF_10_ = .254; 95% CI [.04,.11]) It should be noted that recall was close to floor for all test versions, which was by design, so these results should be interpreted with caution. In terms of recognition performance across all trials (initially recalled and non-recalled trials), there was anecdotal support for the null hypothesis that there was no mean difference between visit order (BF_10_ = .461; P(M|data) = .315; 95% CI [.54,.62]) and test versions (BF_10_ = .605; P(M|data) = .377; 95% CI [.54,.64]). Because of differences in recall between tests, performance was standardized by test version when analyzing the effects of online HD-tDCS on recall performance.

Several Bayesian independent samples t-tests were conducted to assess if participants who received sham over the *left DLPFC* performed differently than those who received sham over the *right DLPFC*. The results showed anecdotal support for the null hypothesis that there were no significant differences between the left and right DLPFC sham condition on average recall (BF_10_ = .498; 95% CI [.02,.14]), recognition (BF_10_ = .408; 95% CI [.45,.65]), FOK ratings (BF_10_ = .432; 95% CI [3.0, 5.5]), and d_a_ (BF_10_ = .340; 95% CI [-.8,.6]). Thus, for all subsequent analyses, sham condition was collapsed across the two montages.

### Lack of effects of online HD-tDCS on memory performance

We tested the effects of stimulation condition on the different memory measures (i.e., recall and recognition). A Bayesian repeated measures ANOVA on recall accuracy, standardized by test version between stimulation conditions, was conducted. Our data showed moderate support for the null hypothesis that there was no difference in recall performance between stimulation groups (BF_10_ = .120; P(M|data) = .107; 95% CI [-.02,.02]). In fact, our data was 8.32 times more likely to occur under the null hypothesis compared to the alternative.

**Fig 3:**
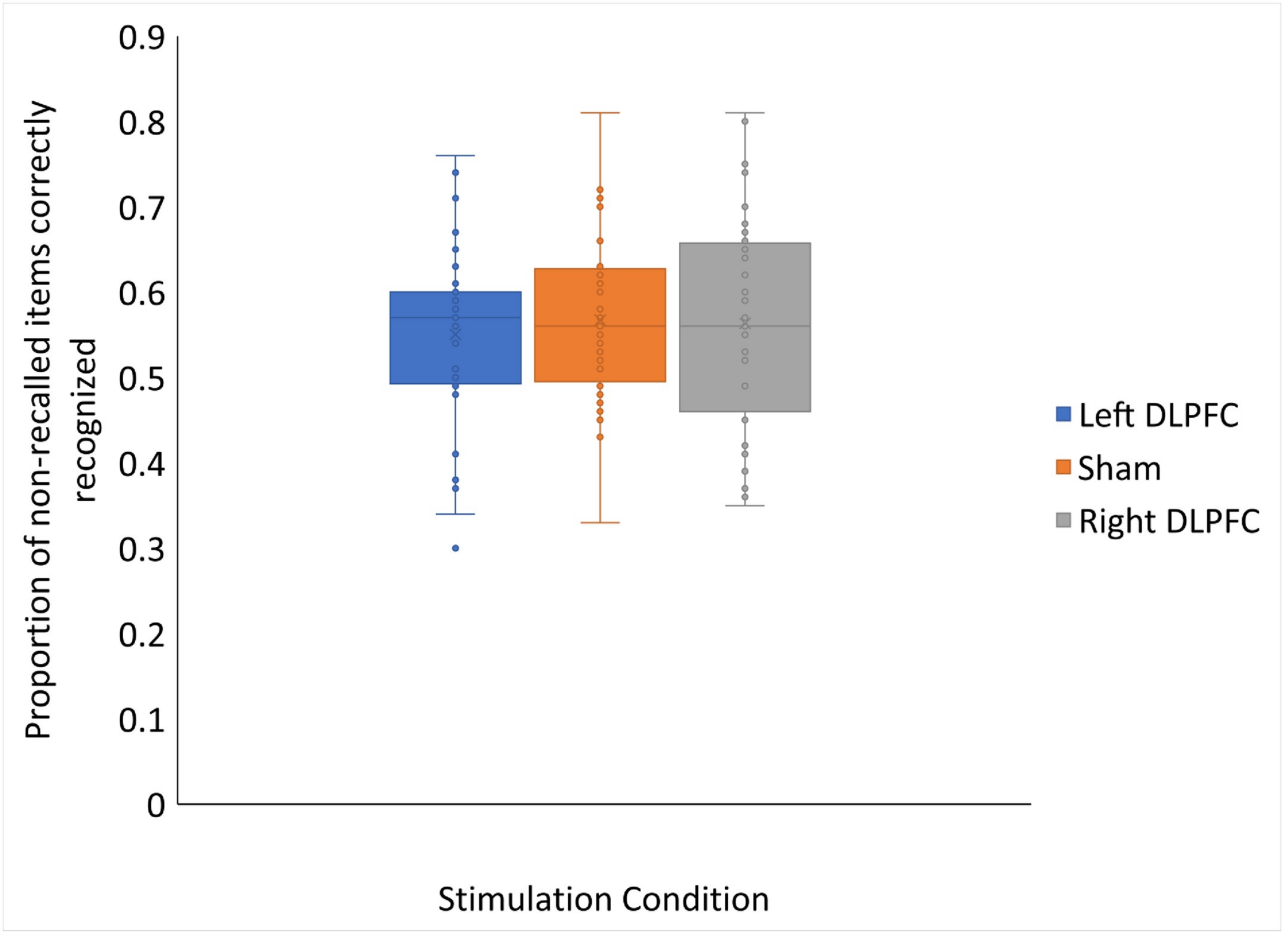
Lack of effects of anodal HD-tDCS over the right or left DLPFC on episodic recognition. There was no difference between stimulation conditions in recognition accuracy for non-recalled trials. Errors bars represent the 95% confidence interval calculated using the inclusive mean.

A Bayesian repeated measures ANOVA was conducted to determine if recognition accuracy differed by stimulation condition for all trials. The results showed moderate support for the null hypothesis that there was no difference in recognition performance between stimulation groups for all trials (BF_10_ = .112; P(M|data) = .116; 95% CI [.52,.62]). In this analysis, our data was 8.96 times more likely under the null hypothesis, showing that stimulation did not affect recognition across all trials. The same pattern of results was found for recognition accuracy for non-recalled trials (See Fig 3), with our data showing moderate support for the null hypothesis (BF_10_ = .137; P(M|data) = .121; 95% CI [.52,.60]), and being 7.28 times more likely under the null.

### Lack of effects of online HD-tDCS on metamemory performance

We next tested the effects of stimulation condition on the different metamemory measures (i.e., FOK ratings and d_a_). A 2 (recognition: correct vs incorrect) x 3 (stimulation condition: *left DLPFC* vs sham vs *right DLPFC*) Bayesian repeated measures ANOVA on average FOK ratings was conducted. Looking at the effect of stimulation on FOK ratings, the results showed strong support for the null hypothesis that there was no difference in average FOK ratings between stimulation conditions (BF_10_ = .070; P(M|data) = 7.60 x 10^−13^; 95% CI [3.20, 4.80]). In fact, our data was 14.31 times more likely under the null hypothesis, showing that stimulation did not affect FOK ratings. As expected, FOK ratings did differ by recognition accuracy, with our data showing extreme support for the alternative hypothesis that there was a significant difference in average FOK ratings between correct and incorrect recognition (BF_10_ = 8.48e^10^; P(M|data) = .922; 95% CI [3.20, 4.80]). This shows that participants had greater average FOK ratings for correct (*M* = 4.46, *SD* = 1.83) compared to incorrect recognition (*M* = 3.71, *SD* = 1.61). Our data also showed very strong evidence for the null hypothesis that there was no interaction between stimulation condition and recognition accuracy on FOK ratings (BF_10_ = .028; P(M|data) = .007; 95% CI [3.20, 4.80]).

**Fig 4:**
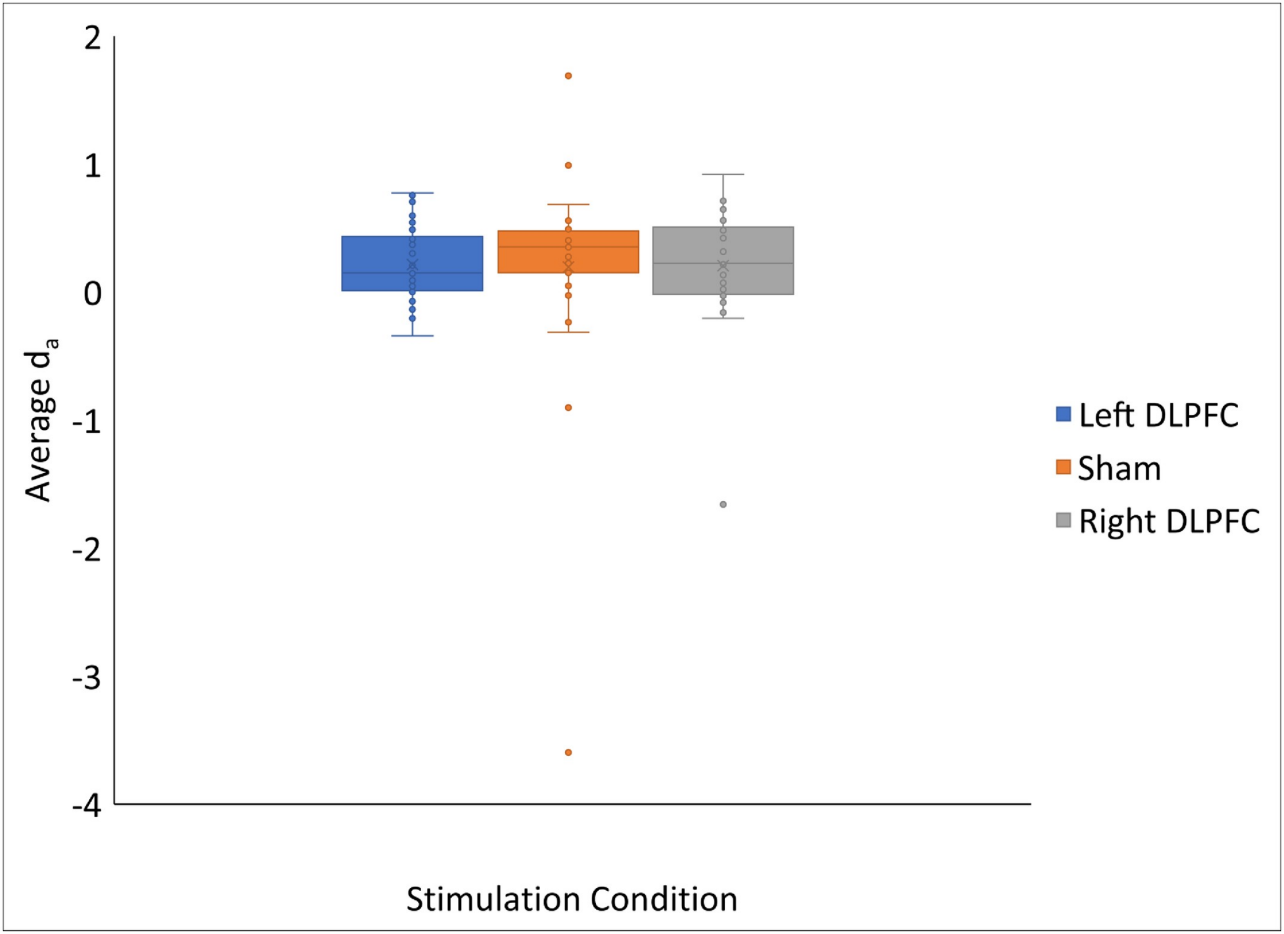
Lack of effects of anodal HD-tDCS over the right or left DLPFC on metamemory monitoring accuracy. Stimulation had no effect on metamemory monitoring accuracy, as quantified by d_a_, for an episodic memory task. Errors bars represent the 95% confidence interval calculated using the inclusive mean.

Finally, we tested if stimulation condition had an impact on metamemory monitoring accuracy, which was quantified by calculating d_a_ between FOK ratings and recognition (incorrectly recalled trials only). Four subjects were removed from this analysis because of an inability to accurately calculate d_a_ due to participants not using enough of the FOK rating scale. The results showed moderate support for the null hypothesis that there is no difference in average d_a_ between stimulation conditions (BF_10_ = .112; P(M|data) = .101; 95% [CI -.10,.40), with our data being 8.94 times more likely under the null hypothesis (see Fig 4). We also conducted this analysis without the presence of one participant who was an outlier in the sham and right DLPFC conditions, which again showed moderate support for the null hypothesis that there is no difference in average d_a_ between stimulation conditions (BF_10_ = .168; P(M|data) = .144; 95% [CI.10,.50), with our data being 5.97 times more likely under the null hypothesis. This shows that stimulation did not have an effect on metamemory monitoring accuracy for this episodic task. For a full summary of the results, see Table 3.

**Table 3:** Means and SDs for different memory and metamemory measures by stimulation condition.

	Left DLPFC	Right DLPFC	Sham
Recall	.07 (.*08*)	.07 (.*08*)	.06 (.*07*)
Recognition	.57 (.*13*)	.59 (.*13*)	.57 (.*14*)
FOK correct recognition	4.43 (*1*.*98*)	4.52 (*1*.*87*)	4.43 (*1*.*93*)
FOK incorrect recognition	3.73 (*1*.*74*)	3.77 (*1*.*71*)	3.63 (*1*.*65*)
d_a_*	.215 (.*29*)	.206 (.*45*)	.196 (.*81*)

* d_a_ is indexed by FOK ratings and recognition for incorrect recall trials.

## Discussion

The primary goal of this experiment was to test the effects of online HD-tDCS over the DLPFC on metamemory monitoring in an associative retrieval task. This work was motivated by prior neuroimaging work showing that activity in the left and right DLPFC correlated with the level of FOK [22] and that HD-tDCS over the left DLPFC improved FOK accuracy in a semantic task [28,29]. In this study, however, online HD-tDCS failed to produce meaningful changes in metamemory monitoring accuracy or associative recognition. The lack of effects may be related to task or stimulation parameters, which warrant careful consideration.

Inconsistent with our predictions, neither online HD-tDCS over the right nor the left DLPFC resulted in changes in memory and metamemory performance compared to sham, and there were no hemispheric differences. The HD-tDCS procedures were the same as in prior work that showed improved metamemory accuracy for a semantic task [28,29] in terms of inclusion and exclusion criteria, training, quality assurance, and location, so we do not think that those factors played a role in the lack of significant effects. The current experiment differed in research personnel and because these studies were all single-blind, we cannot rule out experimenter effects and future studies should employ double-blind designs. Another difference was the comparison of the regions of interest given active stimulation. Prior work compared the left DLPFC to the left anterior temporal lobe (ATL), and showed significant effects, whereas the current study compared the left and right DLPFC and showed no significant effects. This raises the possibility that the comparison between regions played a role in the significance of the effects.

Turning to specific components of the task, in terms of recall, performance was at floor for all stimulation conditions, so it is unlikely we would be able to have seen effects of online HD-tDCS on recall. Note that low levels of recall was by design because the goal was to examine the feeling-of-knowing which occurs after failed recall. Other work has shown that recall was not affected by HD-tDCS over the left DLPFC [29], though when recall difficulty was manipulated, HD-tDCS over the ATL showed effects for medium difficulty items [28]. Thus, regions other than the DLPFC, such as the posterior parietal cortex, may be better suited for improving associative retrieval via HD-tDCS [for a review see– 116]. Alternatively, our results could also indicate that recall was not at the appropriate level of difficulty to show effects of HD-tDCS [55,131,132].

Turning to recognition, perceived task difficulty, motivation, and fatigue may have contributed to the lack of effects for recognition [133–135], and may have had downstream consequences for FOK. Effects of stimulation have been shown to be modulated by task difficulty [131,132], and perhaps the task was too difficult. Even though recognition performance was above chance the chance rate of 33% (57%–59%; See Table 3), anecdotally participants freely reported the task was difficult and thus, task difficulty, or at least perceived difficulty, may have diminished motivation and may have played a role in our null results. Indeed, our post-stimulation questionnaire showed that many participants reported trouble concentrating and sleepiness, and these ratings were higher when informally comparing them to prior work that showed effects of HD-tDCS metamemory monitoring accuracy for a semantic task [28,29]. Prior work has shown that metamemory for too easy or too difficult items is not as predictive as metamemory for medium difficulty items [136,137], and issues related to the memory task difficulty and motivation may have also made it difficult for participants to make FOK ratings predictive of their memory performance.

We also considered whether our results indicated that the DLPFC does not play a causal role in episodic FOK accuracy, unlike semantic FOK accuracy. Conventional tDCS targeting the right and left DLPFC did not show improvements in episodic FOK accuracy either [75]. HD-tDCS could have led to improved metamemory monitoring accuracy for a semantic task [28,29] but not an episodic task due to differences in the cognitive processes required for these two types of memory [138]. Metamemory judgments for semantic and episodic tasks are likely to be based on different sources of information [139], with semantic FOK ratings being based on the retrieval of lexical or semantic information related to the target, and episodic FOKs requiring an autonoetic component, and thus may have been influenced by HD-tDCS differently. However, given the neuropsychological [62–64], fMRI [5,22,23,66,70], and TBS evidence [74], we think a more likely explanation for the lack of effects relates the task being too difficult for HD-tDCS to induce meaningful effects on cognition [28,131,140].

Another possible explanation for our lack of effects is that our stimulation protocol was not sufficient for inducing brain changes that resulted in changes memory and metamemory performance [109,141–143]. Most studies using tDCS have typically used current intensities ranging from 1–2 mA because of a desire to minimize adverse effects [144]. However, it has been shown that tDCS and HD-tDCS doses of 4 mA are well tolerated in healthy adults and patients [50,145–147] and even up to 10 mA in older adults [148]. These higher doses of tDCS may show bigger effects on brain and behavior. Indeed, Shinde et al., [149] showed a linear effect of dose (Sham, 2mA, and 4mA) on regional cerebral blood flow (rCBF) in motor cortex and improvements in a finger sequence task. However, the 4mA condition, but not the 2mA condition, also showed changes in functionally connected frontal regions. Although this work targeted other brain regions, it suggests that higher doses of tDCS are more likely to show larger effects on brain and behavior. Future work could examine dose-response curves and include higher doses of tDCS to determine if the lack of effects seen here were related to the amount of current.

The reporting of null results in tDCS research, while still infrequent, is becoming a more common practice [150–155], which is important for furthering brain stimulation research. Some work suggests that single sessions of tDCS may not be sufficient for inducing behavioral and cognitive changes, and multisession protocols may be warranted [27]. Nevertheless, replication in tDCS is an issue, with some research showing effects of brain stimulation on cognitive processes [28,29,38,156,157], while others have failed to garner significant results [158–160]. The difficulty with replicating tDCS results could be due to a variety of factors, such as inadequate sample sizes, insufficient current, improper electrode placement, task difficulty, and even individual differences in participants [27,55]. Sample size was based on prior work [28,29,75,121] and power analyses, so this should have been a minimal issue. Current and electrode placement was also based on prior work, so impacts should be minimized. However, despite replicating the procedure, individual variability in head and brain structure may have contributed to the lack of effects [41,161]. There is also work showing that tDCS efficacy can depend on structural properties of neurons, such as the size, location, how the neuron is oriented to the current being applied, and even the type of neuron [45]. In some cases, anodal tDCS can be inhibitory, leading to an opposite cognitive or behavioral effect than originally hypothesized. To account for this in future studies, future work could consider individually tailored tDCS [162,163]. TDCS efficacy can also differ based on state-dependent factors such as the time of day [32], or participant arousal level [151]. Prior work showed that tDCS was most effective in improving episodic retrieval during the morning, which is considered a non-optimal cognitive processing time in the younger adult population [32]. Individual participants received HD-tDCS at approximately the same time for each visit, but different participants came at different times of the day, which could explain why no effect of HD-tDCS was found for the episodic task. Future research should aim to hold time of day constant between all participants to maximize the effects of tDCS. Also, as previously stated, the participants in the current study may have had a lower level of arousal, as shown through the high reports of sleepiness and trouble concentrating, which could have modulated the effects of HD-tDCS on cognition. It could have been that our stimulation protocol (i.e., current, montage, task demands) were insufficient to induce effects of cognition, though more work identifying the conditions in which tDCS effects cognition is needed. Collectively, this indicates that more systematic testing of the parameters in which tDCS does and does not work is needed.

Recently, there has been a call for reporting Bayesian statistics in brain stimulation studies [125]. An argument can be made that small sample sizes or single stimulation sessions are not enough to show an effect of stimulation, and that just because null results are found, this does not mean that tDCS and other forms of brain stimulation are ineffective. Bayesian analyses compare the data in terms of both alternative and null hypotheses, and can indicate whether the protocol was unsuccessful, or if there is not enough evidence to conclude that the protocol didn’t work. In the case of this experiment, the results from Bayesian analyses showed moderate support for the null hypothesis, indicating that our protocol was unsuccessful in finding HD-tDCS related differences in memory and metamemory. However, these null results do not invalidate HD-tDCS as a useful tool in altering cognition across the board. Instead, more research into the exact mechanisms of tDCS is needed, as well as a more consistent protocol for applying stimulation. In fact, some researchers believe that stimulation should be individualized to the person to garner maximum effects, and have used computer modeling to test the efficacy of individualized stimulation protocols [40,164]. This work is still in the early stages of development, but one day, could prove to be a useful tool in HD-tDCS research.

One limitation that should be noted is the use of single blinding in this experiment. Despite participants being blind to stimulation condition, the researchers were not, which could have led to demand characteristics being imposed on participants. However, an analysis utilizing Cochran’s Q and participants perceived stimulation showed that participants were blind to active versus sham, and thus we do not believe that this impacted our results. It’s also important to note that stimulation was aborted manually, which, in the case of sham, meant that the current did not ramp back up to 2 mA and then down to 0 mA to simulate sensations felt during active stimulation. However, we do not anticipate that this was an issue, as participant blinding was shown to be adequate.

## Conclusions

Despite the lack of effects of HD-tDCS over the left or right DLPFC on episodic memory retrieval and metamemory monitoring, we do not endorse the idea that the DLPFC is not involved in memory and metamemory monitoring processes. Bayesian analyses showed moderate support for the null hypothesis, showing our tasks did not elicit HD-tDCS related differences in memory and metamemory. Our results suggest that either our task was too difficult to distinguish meaningful effects, or that our stimulation parameters were not sufficient to result in HD-tDCS induced changes in memory and metamemory monitoring accuracy. Results from studies assessing the effects of tDCS on episodic memory are mixed [101,109,111,113], and thus future research should attempt to elucidate the differences between successful and unsuccessful tDCS protocols. Reporting these null results are important for allowing for better experimental design in future research and allowing a better estimate of the effects of tDCS. We suggest that future work carefully consider task difficulty and stimulation parameters to better understand the potential use of HD-tDCS as an intervention. There is a need to determine the effects of different current intensities on episodic memory, as results from studies assessing other memory processes show that lower intensities may be more effective compared to higher intensities [53,56]. It could be that individualized doses of current are needed to modulate cognitive processes [165], as individual differences, such as sex [141], and even time of day [32], have been shown to modulate the effects of tDCS. However, more work is needed to standardized current intensity titration protocols. Others have suggested determining individualized dosages of current by using magnetic resonance imaging (MRI) to model current flow for each subject [166,167], though this process is both expensive and time consuming. Future work should focus on how to best individualize tDCS protocols based on factors that could modulate stimulation efficacy.

## Supporting information

S1 File Data file for analyses presented in the paper. Usage instructions and limitations are in the file.
(XLSX)
